# HLA-DPB1 Variant Effect on Hepatitis B Virus Clearance and Liver Cirrhosis Development Among Southwest Chinese Population

**DOI:** 10.5812/hepatmon.19747

**Published:** 2014-08-18

**Authors:** Zhangyong Hu, Jun Yang, Guolian Xiong, Han Shi, Yuan Yuan, Lin Fan, Yali Wang

**Affiliations:** 1 Department of Infection Diseases, First Affiliated Hospital, Chengdu Medical College, Chengdu, China

**Keywords:** Hepatitis B virus, Single Nucleotide Polymorphism, Human, Leukocyte, Antigen

## Abstract

**Background:**

Previous studies have shown that genetic variants in HLA-DP genes affect disease progression in hepatitis B virus (HBV) infection.

**Objectives:**

We aimed to evaluate possible association between HLA-DPB1 rs9277534 polymorphism and different clinical complications of hepatitis B virus (HBV) infection.

**Materials and Methods:**

Snapshot assay was used to investigate the association of rs9277534 polymorphism in 342 patients with persistent HBV infection and 342 age and gender-matched HBV spontaneous clearance controls. Patients were categorized into asymptomatic HBV carriers (AsC, n = 104), chronic hepatitis B (CHB, n = 116), and liver cirrhosis (LC, n = 122) subgroups.

**Results:**

There was a significantly higher proportion of the rs9277534 minor allele A in HBV spontaneous clearance control than that in HBV persistent infection group (OR = 0.58, 95%CI = 0.46-0.73, P < 0.0001). Genotypic analysis showed that GA and AA genotypes were associated with HBV spontaneous clearance (GA: OR = 0.56, 95%CI = 0.40-0.79, P = 0.019; AA: OR = 0.24, 95%CI = 0.14-0.44, P < 0.0001). A significant difference was found between AsC and LC groups in the distribution of AA genotype (OR = 9.32, 95%CI = 1.293-67.14, P = 0.027).

**Conclusions:**

Variant at rs9277534 could affect both the spontaneous clearance of HBV infection and progression from asymptomatic HBV carriers to HBV-related liver cirrhosis in Southwest Han Chinese population.

## 1. Background

Hepatitis B virus (HBV) infection results in various clinical complications including HBV spontaneous clearance, asymptomatic HBV carrier (AsC), chronic active hepatitis B (CHB), fulminant hepatitis, liver cirrhosis (LC), and even hepatocellular carcinoma (HCC) (1). Although the exact mechanisms of HBV persistence are still not fully defined, a complex interplay of viral, environmental, and genetic components including age, gender, viral genotype, host immunity, and infection with other viruses are thought to be associated with persistent HBV infection (2, 3). However, host immune response against HBV is believed to be the most important determinant of different outcomes after HBV infection. Numerous studies suggested that single nucleotide polymorphisms (SNPs) in host gene could correlate with persistence of HBV infection or HBV spontaneous clearance. A genome-wide association study emphasized the importance of human leukocyte antigen (HLA) SNPs in Japanese and Thai patients with chronic hepatitis B; subsequently, significant associations were reported in other Asian countries (4-10). However, Thomas et al. (11) reported that HLA-DPB1 rs9277534 was significantly associated with HBV recovery in both African-American and European populations; while HLA-DPB1 rs9277535, which strongly associated with chronic hepatitis B infection in Asia, only had a marginal effect on HBV infection in African-American and European populations. In contrast, the HLA-DPB1 rs9277535 SNP was not associated with HBV infection in Caucasians (12). Though there was a significant association with persistent HBV infection in some studies, results are contradictory regarding the correlation of HLA-DP SNPs and progression of chronic HBV infection (13, 14).

## 2. Objectives

The present study involved the selection of rs9277534 within HLA-DPB1 gene to identify the association with different clinical complications after HBV infection in Han Chinese population from Sichuan province of Southwestern China.

## 3. Materials and Methods

### 3.1. Ethics Statement

The research protocol was reviewed and approved by the Ethics Committee of the First Affiliated Hospital of Chengdu Medical College in Sichuan province of Southwestern China. Written informed consent was obtained from all participants, and all samples were blinded.

### 3.2. Study Population

Three hundred and forty-two unrelated Chinese Han patients with persistent HBV infection were enrolled from the First Affiliated Hospital of Chengdu Medical College. Of which, 104 were chronic asymptomatic HBV carriers (AsC), 116 patients had active chronic hepatitis B (CHB) infection, and 122 had liver cirrhosis (LC). Persistent HBV infection was defined to have positive results for HBsAg and anti-HBc for at least six months with or without HBeAg positivity. Clinical diagnoses in patients were performed based on the Guideline of Prevention and Treatment for CHB (Chinese Society of Hepatology and Chinese Society of Infectious Diseases, Chinese Medical Association, 2010). A group of 342 age- and sex-matched individuals with spontaneous clearance of HBV (HBV-resolved) enrolled as the control group. HBV-resolved was defined as individuals who spontaneously recovered without treatment and with negative results for HBsAg, HBeAg, and HBV deoxyribonucleic acid (DNA), but positive results for antibody to hepatitis B surface antigen (anti-HBs) and antibody for hepatitis B core antigen (anti-HBc). All individuals were tested to exclude hepatitis C (HCV), hepatitis D (HDV), and human immunodeficiency virus (HIV) infections.

### 3.3. Extraction Genomic Deoxyribonucleic Acid and Polymorphisms Genotyping

Genomic DNA was extracted from peripheral blood leukocytes using TIANamp Blood DNA kit (Qiagen, Hilden, Germany) following the manufacturer's instructions. The concentration and purity of DNA were tested by NanoDrop spectrophotometer (Thermo Fisher Scientific, Wilmington, DE, USA) and DNA was diluted to a final concentration of 10ng/μL. Genotyping was performed by SNaPshot method, described in detail in a previous study (15). The sequence of primers for genotyping was as follow:

rs9277534F: CAAATCAAGTTTAGTGCCCTCA;

rs9277534R: GCTGCTGTGCACTGATTGTGTG; 

rs9277534SR:ttttTTTTTTTTTTTTTTTTTTTTTTTTTTTTTTTTTTTAGTCTGCTCACCATTGAATAGTTAAGAATAG.

Data was analyzed by GeneMapper 4.1 (Applied Biosystems, USA). Repeated genotyping in 10% of randomly selected samples yielded 100% identical results.

### 3.4. Statistical Analysis

Assumption of Hardy–Weinberg equilibrium was tested for the rs9277534 SNP using DeFinetti program (http://ihg.gsf.de/cgi-bin/hw/hwa1.pl). All statistical analyses were conducted using SPSS for Windows (version 18.0; SPSS Inc. Chicago, IL, USA). Chi-square-test was used to compare distribution of genotypes between patients and controls. Odds ratios (ORs) with their 95% confidence intervals (CIs) were calculated based on the binary logistic regression analysis and were adjusted for age and gender. P value < 0.05 was considered as statistically significant.

## 4. Results

### 4.1. Subjects Characteristic and Hardy-Weinberg Equilibrium

A total of 342 patients with persistent HBV infection (165 males and 177 females) and 342 age- and sex-matched HBV-resolved controls were enrolled in this study. Baseline characteristics are shown in Table 1. The genotypic distributions of SNP were in accordance with the Hardy-Weinberg equilibrium in the both groups.

**Table 1.** Clinical Characteristics of Study Subjects ^a^

variable	Patients (n = 342)			Control (n = 342)
	ASC, (n = 104)	CHB, (n = 116)	LC, (n = 122)	
**Gender**				
Male	22	51	92	165
Female	82	65	30	177
**Age, y, Mean **± **SD**	35.5 ± 12.4	37.0 ± 14.6	50.6 ± 11.0	41.6 ± 14.5
**HBsAg+**	104	116	122	0
**Anti-HBs+**	0	0	0	289
**Anti-HBc+**	99	107	113	309

^a^ Abbreviations: AsC, Asymptomatic HBV carriers group; CHB, Chronic active hepatitis B group; LC, HBV-related liver cirrhosis group.

### 4.2. Genotypic and Allele Frequencies of rs9277534

The minor alleles (A allele) of rs9277534 were observed at frequencies of 27.78% in patients with HBV and 39.77% in controls, respectively. In the case of G allele as conference, the A allele carriage was significantly associated with HBV spontaneous clearance (OR = 0.58; 95% CI: 0.46, 0.73; P < 0.0001). There were significant differences between case and control groups in genotype distributions. Compared to individuals carrying wild-type GG genotype of rs9277534, those with AA or GA genotype had a decreased risk of persistent HBV infection (OR = 0.24; CI: 0.14, 0.44; P < 0.0001 for AA; OR = 0.56; CI:0.40,0.79; P < 0.0001 for GA), indicating a plausible protective role for AX genotype against HBV (Table 2).

**Table 2.** Genotype and Allele Frequencies of rs9277534 in Persistent HBV Infection and Hepatic B Virus-Resolved Group^a,b^

rs9277534	Case Group, (n = 342)	Control Group, (n = 342)	OR (95%CI)	x^2^	P Value
**Genotype**					
GG	176 (51.5)	123 (36.0)	1	17.94	< 0.0001
GA	142 (41.5)	166 (48.5)	0.56 (0.40-0.79)	5.52	0.019
AA	24 (7)	53 (15.5)	0.24 (0.14-0.44)	21.68	< 0.0001
G	494 (72.22)	412 (60.23)	1	21.98	< 0.0001
A	190 (27.78)	272 (39.77)	0.58 (0.46-0.73)		
**Dominant model**					
GG	176 (51.5)	123 (36.2)	1		
GA+AA	166 (48.5)	219 (63.8)	0.45 (0.33-0.61)	37.38	< 0.0001

^a^ Abbreviations: OR, odds ratio; 95% CI, 95% confidence interval; GG, G/G genotype; GA, G/A genotype; AA, A/A genotype.

^b^ Case group, persistent HBV infection; control group, HBV-resolved group.

### 4.3. rs9277534 Polymorphism in Different Persistent Hepatic B Virus Infection Subgroups

To determine whether rs9277534 polymorphism was associated with progression of persistent HBV infection to liver cirrhosis, a similar analysis was performed by comparing LC with AsC group, CHB with AsC group, and LC with CHB group. Unexpectedly, the frequency of rs9277534 AA genotype was significantly higher in LC group than AsC group (OR = 9.32; CI: 1.29, 67.14; P = 0.027), suggesting that patients carriage AA genotype had a higher risk for developing liver cirrhosis (Table 3). However, there were no significant differences between CHB and LC groups (OR = 3.19, CI: 0.84, 12.08; P = 0.09) after adjustment for age and sex.

**Table 3.** Genotype frequencies of HLA-DPB1 rs9277534 polymorphism in AsC, Chronic Active Hepatitis and Liver Cirrhosis Group^a^

Genotype	Control, No. (%)	Case, No. (%)	Adjusted OR (95%CI)	P Value
**AsC vs. CHB**				
GG	58 (55.8)	64 (55.2)	1	0.70
GA	43 (41.3)	47 (40.5)	0.99 (0.56-1.74)	0.97
AA	3 (2.9)	5 (4.3)	1.90 (0.41-8.69)	0.41
GA+AA	46 (44.2)	52 (44.8)	1.02 (0.54-1.94)	0.96
**AsC vs. LC**				
GG	58 (55.8)	54 (44.3)	1	0.06
GA	43 (41.3)	52 (42.6)	0.81 (0.36-1.81)	0.61
AA	3 (2.9)	16 (13.1)	9.32 (1.29-67.14)	0.027
GA+AA	46 (44.2)	68 (55.7)	1.16 (036-3.71)	0.81
**CHB vs. LC**				
GG	64 (55.2)	54 (44.3)	1	0.22
GA	47 (40.5)	52 (42.6)	1.01 (0.52-1.97)	0.98
AA	5 (4.3)	16 (13.1)	3.19 (0.84-12.08)	0.09
GA+AA	52 (44.8)	68 (55.7)	1.66 (0.58-4.71)	0.34

^a^ Abbreviations: AsC, Asymptomatic HBV carriers; CHB, chronic active Hepatitis B; GG, G/G genotype; GA, G/A genotype; AA, A/A genotype; LC, liver cirrhosis.

## 5. Discussion

Classical human leukocyte antigen loci are classified into two types of class I (HLA-A, B, C, E, F, G, H, and J) and class II (HLA-DR, DP, DQ, DO, and DM) molecules. Following their discovery in 1970s, HLA gene was selected as the major gene to study the susceptibility to infectious diseases (16). HLA-DPA1 and HLA-DPB1 molecules, the central components of MHC class II, are involved in antigen presenting to CD4+ T helper cells. Therefore, they are very crucial to induce immune response against infectious diseases (17). Accumulating evidences suggested that HLA-DP variants are related to liver diseases. Hirayama et al. (18) confirmed that the HLA-DR-DQ alleles had a protective effect on early changes of liver fibrosis, while the HLA-DP alleles had a protective role against the late phase of schistosomal hepatic fibrosis. Zhang Q et al. (19) reported that HLA-DP could affect cirrhosis and HCC risks through interacting with HBV mutants. Guo X et al. (20, 21) demonstrated that genetic variants in the HLA-DP locus were strongly associated with persistent HBV infection in both Northern and Southern Han Chinese population, but not with HBV progression. O’Brien TR et al. (22) found that rs3077 and rs9277535 were significantly associated with decreased mRNA expression of HLA-DPA1 and HLA-DPB1, respectively. It had been hypothesized that lower expressions of HLA-DPA1 and HLA-DPB1 could be risk predictors of chronic HBV infection. In the present study, there was a significant difference between patients with persistent HBV infection and HBV-resolved controls at both allele and genotype levels, suggesting the rs9277534 minor A allele as a protective factor against HBV infection, which was consistent with the previous study. In addition, stratified analysis by different chronic HBV infection status revealed that there was a significant difference in genotypic frequencies distribution between patients with AsC and LC. Unexpectedly, LC group had a higher frequency of genotype AA than AsC group after age and sex adjustment. Hence, it could be considered that the HLA-DP rs9277534 variant may not only be involved in the process of HBV spontaneous clearance, but it may also be related to the progression of CHB to end-stage liver disease in Southwest Han Chinese population. Effective T cell response plays a critical role in HBV clearance, and inadequate HBV-specific cytotoxic T cell (CTL) responses are assumed to be the leading cause of HBV persistent infection (23). Thomas R et al. (11) demonstrated that the rs9277534 GG genotype healthy donors had higher levels of HLA-DP surface protein in PBMCs and intracellular transcript level expression than individuals carrying non-GG genotypes; perhaps higher HLA-DP expression could promote Th2 dominant immune response along with poor CTL activity in patients with HBV infection, thus resulting in HBV persistence. While in patients with chronic HBV infection, lower level of HLA-DP protein expression in individuals bearing AA genotype might favor Th1 responses; therefore, on one hand, repeated liver cell injury mediated by inadequate Th1 responses to HBV infection might increase the risk of HBV-related liver cirrhosis and hepatocellular carcinoma. On the other hand, Th1 dominant immune responses associated with polyclonality and multispecificity of HBV-specific CTL responses could completely clear HBV. This assumption might support previous finding revealed by Cheng HR (24) indicating that patients with CHB bearing non-GG genotype of rs9277535, which was reported to be in perfect linkage disequilibrium with SNP rs9277534 in 3'UTR of HLA-DPB1 gene in Chinese population, had higher chance of spontaneous HBsAg seroclearance. In conclusion, the present study showed that carriers with rs9277534 A allele and AA genotype were more likely to clear HBV spontaneously; while in patients with persistent HBV infection, AA genotype could be related to progression of chronic HBV carriers to liver cirrhosis. However, some limitations still exist in this study. Firstly, virus factors such as virus mutants and HBV DNA level were not taken into consideration while evaluating the risk of cirrhosis development. Secondly, a relatively small sample in each subgroup was studied which could lead to insufficient power for statistical analyses. In addition, previous studies concerning the influence of HLA-DP variants on gene expression were conflicting (11, 22). Therefore, further studies including a large number of samples are necessary to validate the findings of this study to clarify the potential functions of rs9277534 in HBV infection.
